# RAD: A Paradigm, Shifting

**DOI:** 10.1093/biosci/biab123

**Published:** 2021-11-17

**Authors:** John W Williams

**Affiliations:** Department of Geography, University of Wisconsin-Madison, Madison, Wisconsin, United States

For any ecosystem manager working today, it is virtually certain that, over their entire professional career, global mean temperatures will continue to rise, with various cascading effects on the hydrosphere, the cryosphere, and the biosphere. Rainfall variability is intensifying, with net increases in water availability in some regions and aridification in others, often in areas where water is already scarce. Sea level is rising, causing saltwater encroachment in coastal ecosystems and increased damage from hurricanes and other coastal storms. Phenological events are changing in timing, species are changing in abundance and distributions, novel mixtures of species are emerging, and wildfire regimes are intensifying. As a result, ecosystems are beginning to transform (Jackson 2021), just as the Earth's biosphere was massively ­transformed by a 6 °C global ­warming at the end of the last ice age (Nolan et al. 2018).

In response, the theory and operational practice of ecosystem management is undergoing a paradigm shift. The phrase *paradigm shift* can be overused, but for ecosystem managers, the implications of climate change are profound. Climate stationarity is dead (Milly et al. 2008), the climate system is experiencing persistent directional change toward a state with no precedent in human history (Burke et al. 2018), and management policies that assume a stable baseline no longer apply. As Magness and colleagues (2021, this issue) write, ecosystems and environments are “shifting from historical baselines that are generally observable, knowable, and agreed on to nonstationary conditions that are novel, uncertain, and contested.” Given this, what is a manager to do?

This special issue of *BioScience* represents a milestone in this paradigm shift. The articles here present and explore a simple but comprehensive operational framework, called RAD (resist–accept–direct), representing three basic options that fully encompass the decision space available to managers. Managers can resist change, seeking to keep ecosystems in a current state, or at least slow the rates of transformation. Managers can accept change, letting changes proceed with minimal intervention. Or managers can seek to direct change, steering ecosystem transformations toward desired and away from undesired outcomes. RAD discards the prior working assumptions that ecosystems and environments are inherently stable within some historical baseline of variation (Schuurman et al. 2021, this issue) and that a central management goal is to restore systems to historical baseline states. RAD instead adopts the working assumptions of ongoing directional environmental change and the likelihood of ecosystem transformations at rates that can be fast, slow, or abrupt (Williams et al. 2021). (Also discarded by RAD is the concept of resilience, which too often focuses on the capacity of an ecosystem to maintain or return to a prior baseline state.)

At one level, the RAD framework is not new. Many papers have called for a rethinking of management policy in the face of climate change—­notably, Millar and colleagues (2007), who presented a tripartite framework (resistance–resilience–response) that was a foundational step toward RAD. See also Aplet and Cole (2010) for another early description of the tripartite framework and Hunter and colleagues (1988) for an early thought piece on how to preserve biological diversity under changing climates. One of the strengths of RAD is that it closely builds on these prior frameworks, with Schuurman and colleagues (2021, this issue) providing a thoughtful review of RAD and its intellectual history.

Yet to simply view RAD as a new gloss on older ideas would be to miss how deeply RAD is now permeating the community of ecosystem managers and institutions, particularly at the federal level. RAD marks the inflection point at which climate change adaptation concepts are moving out of the realm of academic ideation and into institutionalized frameworks for action. The author teams represent a constellation of scientists drawn from across agencies, particularly the US Geological Survey, the National Park Service, and the US Fish and Wildlife Service. Moreover, each of the articles explores some dimension of how RAD can be integrated into and update current management practice, with multiple exemplar systems. For example, Crausbay and colleagues (2021, this issue) establish priorities for scientific questions for supporting manager decision-making, whereas Clifford and colleagues (2021, this issue) explore the internal and external factors that affect how a manager might choose among RAD options, positioning RAD at the nexus between social and ecological systems. Magness and colleagues (2021, this issue) provide foundational principles for ecosystem management in a RAD framework, using the Kenai Peninsula as an example, whereas Lynch and colleagues (2021, this issue) show how the classic adaptive management cycle can be adapted (and complexified) to include RAD.

No single RAD solution fits all. Many managers will likely employ a portfolio of RAD solutions, ­employing, for example, resistance strategies for ecosystems of high cultural or biological importance, such as the ethnographic landscapes surrounding Devils Tower (Lynch et al. 2021, this issue); directing transitions when alternative ecosystem outcomes vary in desirability (e.g., Acadia National Park; Crausbay et al. 2021, this issue); or simply accepting changes while monitoring them and being ready to shift strategies if needed (Lynch et al. 2021, this issue). In the face of uncertainty, the experiments of today will be the solutions of tomorrow, so managers should be ready to try out different management approaches in different portions of their managed landscapes. For any given system, a manager might opt to change strategies over time—for example, first resisting changes for as long as feasible, then accepting changes after a regime shift has taken place, then directing changes after the possibility emerges for alternate ecosystem outcomes, with different levels of desirability to managers and stakeholders.

I had only a few quibbles when reading these articles. As a paleoecologist, I'm always dubious about the word *irreversible*, which some of these authors use freely. Calling an ecosystem transition *irreversible* usually carries implicit assumptions about timescale, environmental forcing, and effort that should be carefully checked. Given enough time, effort, and environmental forcing, almost every ecosystem change can be reversed, barring extinction. Moreover, calling some ecosystem transitions *irreversible* misses the deeper resilience of the biosphere to the many past climate changes and carbon cycle disruptions that have occurred throughout Earth's history.

Note too that, even if anthropogenic climate change wasn't a thing, the paradigm of stationary historical baselines was long overdue for the scrapheap. Viewing the past as stable was always only a convenient assumption. Climates vary across all timescales (von der Heydt et al. 2021), as do ecosystems (Delcourt and Delcourt 1988). Contemporary species and biodiversity distributions carry legacies of the last ice age (Svenning et al. 2015), and North American ecosystems were steadily changing even prior to European arrival and anthropogenic climate change (Dawson et al. 2019).

Moreover, the old paradigm of North America as a pristine wilderness was a dehumanizing one that missed the many and varied interactions of Indigenous societies with their environments and ecosystems over millennia (Fletcher et al. 2021). It is heartening to see many of these essays calling for closer engagement with Indigenous communities and practitioners, although more groundwork is needed to build the close partnerships between Western and Indigenous collaborators that mark successful transdisciplinary climate adaptation science (Smithwick et al. 2019). With RAD, North American ecosystem management frameworks may now come into closer alignment with European approaches today, which are rooted in the recognition that European ecosystems have been repeatedly transformed by human action over the last several thousand years. European conservationists have long been more ready to experiment with forward-looking interventions such as rewilding (Perino et al. 2019), while North American conservationists have usually followed more of a preservationist or restorationist ethos.

Finally, in reading these articles, I was struck by the odd mix of hope and sadness that comes with contemplating climate-driven ecosystem transformations. Hope, because these papers demonstrate how, as climates change, we humans can change too. We can adapt our strategies and optimize our solutions. It is so beguilingly easy, when contemplating the most severe outcomes of climate change, to focus on the most alarming or disruptive outcomes—the losses of coral reefs worldwide, the wildfires that rage in the western US or Australia—and thereby drift into the false refuge of despair, imagining that climate change means the end of life on Earth. But the reality is far harder and far more complex. Life will continue as climates change, and in all likelihood, we humans will continue too. But, sadness, because many of the places we cherish will change. And we will be faced with hard choices: what to save, what to let go, what to fight for even knowing that we may lose that fight. RAD provides a framework for these choices and offers us a way to be proactive instead of reactive, to direct change and to help accelerate species’ natural adaptive capacity to climate change. Helping species, ecosystems, and societies adapt to climate change is a worthy effort; it can be our generation's gift to the future.

## Author Biographical

John W. Williams (jwwilliams1@wisc.edu) is affiliated with the Department of Geography at the University of Wisconsin-Madison, in Madison, Wisconsin, in the United States.
